# Impact of feed additives and host-related factors on bacterial metabolites, mucosal integrity and immune response in the ileum of broilers

**DOI:** 10.1007/s11259-023-10135-9

**Published:** 2023-05-09

**Authors:** Yada Duangnumsawang, Jürgen Zentek, Wilfried Vahjen, Joan Tarradas, Farshad Goodarzi Boroojeni

**Affiliations:** 1 Institute of Animal Nutrition, Department of Veterinary Medicine, Freie Universität Berlin, Berlin, Germany; 2 Faculty of Veterinary Science, Prince of Songkla University, Hatyai, Songkhla Thailand; 3 ‡Institute for Food and Agricultural Research and Technology IRTA, Constantí, Spain

**Keywords:** Goblet cell, Host-microbiota interaction, Immune response, Phytobiotic, Probiotic

## Abstract

The present study aimed to investigate the effect of age, breed, and sex of broilers, as well as a probiotic or phytobiotic product on mucosal morphology, bacterial metabolites, and immune traits in the ileum of broilers. A total of 2,880 one-day-old male and female broiler chicks from two breeds (Ross308® and Cobb500®) were randomly assigned to 72 pens. Broilers were offered a wheat-soybean diet without (CO), or with either a probiotic (PO; 2.4 × 10^9^ CFU/kg of *Bacillus subtilis* DSM32324 and DSM32325 and *B. amyloliquefaciens* DSM25840) or a phytobiotic (PY; grape extract, 165 ppm procyanidin and 585 ppm polyphenols of the diet) product. The trial was conducted with a 3 × 2 × 2 factorial arrangement of diet, breed, and sex in a completely randomized design (6 replicate-pens per treatment). At day 7, 21, and 35, one chicken per pen was slaughtered for collecting ileal tissue to evaluate of histomorphology and mRNA expression, as well as ileal digesta to measure bacterial metabolites. Data were subjected to ANOVA (the main factors; age, diet, breed, and sex) and Four-Way ANOVA (interactions) using GLM procedure. Overall, the concentration of acetate and total short chain fatty acids reached the peak and lactate decreased to its lowest on day 21, but their concentrations at day 7 and 35 were similar (*p* > 0.05). Spermine, spermidine, and ammonia decreased after day 7, while putrescine and cadaverine increased after day 21 (*p* < 0.05). mRNA expression of cytokines, mucin 2 (*MUC2*) and claudin 5 (*CLDN5*) was similar; increased from day 7 to 21 and decreased afterward (*p* < 0.05). Villus height, crypt depth and villus surface area increased with age (*p* < 0.05). Acidic goblet cells (GC) number and density increased after day 21 (*p* < 0.05). Ross broilers showed higher D-lactate concentration and *IFN*-γ expression, while Cobb broilers had greater *IL-4*, *IL-6* and *TNF-α* expression and higher total GC number (*p* < 0.05). Female displayed higher villus height and GC number and density (mixed and total GC) than male (*p* < 0.05). The effect of dietary treatment was not found on any investigated variables (*p* > 0.05). In conclusion, aging of broilers affected ileal histomorphology, cytokine expression, and barrier integrity, as well as bacterial activity. These observed impacts could be attributed to host-microbiota interaction and the direct effects of bacterial metabolites on intestinal cells and immune system.

**Supplementary Information:**

The online version contains supplementary material available at 10.1007/s11259-023-10135-9.

## Introduction

The prohibition of antibiotic growth promotors (AGP) has put tremendous pressure on the poultry industry to look for reliable alternatives. As a result, probiotics and phytobiotics have been widely used to reduce the use of AGP in poultry production. Probiotics are used to regulate intestinal microbiota and can directly influence gut immune system through the pattern-recognition receptors (PRRs) present in both epithelial and immune cells of the host (Tarradas et al. 2020). Spore forming bacteria are resistant to environmental stresses like heat, disinfectants and low pH. *Bacillus* based probiotics have demonstrated superior stability and viability in both feed processing and the gut of broilers compared with non-spore-forming probiotics, making them one of the best probiotic candidates for poultry nutrition (Goodarzi Boroojeni et al. 2016; Zentek and Goodarzi Boroojeni 2020). Feeding *B. subtilis* and *B. amyloliquefaciens* to broilers have been shown to reduce proliferation of pathogens, modify gut microflora, minimize gut inflammation and modify mucosal morphology which finally led to an improved growth performance (Park et al. 2020; (Wang et al. 2021a).

Phytobiotics are plant-based, naturally occurring substances which promote health-related benefits (Chamorro et al. 2019). The beneficial impacts of phytobiotics are attributed to their bioactive compounds including polyphenols which exhibit antioxidant, anti-inflammatory, and antibacterial effects (Viveros et al. 2011). Procyanidins are the main polyphenols found in grape extract and known to reduce pro-inflammatory responses and epithelial damages caused by oxidative stress in the small intestine of broilers (Yang et al. 2017a; Cao et al. 2020). Grape procyanidins can be metabolized by the intestinal microbiota into phenolic acids and other metabolites that help reducing oxidative stress and inflammation in gut of broilers (Chamorro et al. 2019; Cao et al. 2020). Furthermore, addition of procyanidin-rich grape extract to broiler diet has been shown to increase populations of some beneficial bacteria such as *Enterococcus*, whereas it decreased the number of *Clostridium* in the ileum of broilers (Viveros et al. 2011).

The ileum is the terminal part of the small intestine that plays a key role in nutrient absorption. However, in comparison to the proximal part of the small intestine, it appears to be an important site for microbial fermentation as evidenced by an increase in bacterial density and metabolite production distally along the small intestine (Rehman et al. 2007). Gut microbiota directly interacts with intestinal epithelial cells, communicates with immune cells and modulates cell proliferation and barrier function (Mahapatro et al. 2021). Intestinal microbiota of newly hatched broiler chicks is known to have a limited diversity. However, it undergoes successional changes over time and tends to become more diversified and stabilized as the host ages (Glendinning et al. 2019). Dramatic changes in bacterial community composition and activity have been shown to occur naturally as broilers mature (Oakley et al. 2014; Duangnumsawang et al. 2022). However, the direction of these changes seems to be affected by different factors such as intestinal morphology and environment condition provided by the host (Bindari and Gerber 2022).

It has been reported that ileal microbial composition as well as its physiological functions were affected by broiler genotype (Emami et al. 2022) and sex (Lumpkins et al. 2008). Modern broilers are genetically selected for performance and immunocompetence, yet the immunological responses to certain challenges greatly vary between breeds (Jang et al. 2013). On the other hand, distinct gut morphology, such as villus height and crypt depth as well as the mucin composition of the intestinal mucus layer may provide a specific niche for intestinal microbiota, contributing to a breed-specific bacterial community (Mabelebele et al. 2017; Richards-Rios et al. 2020). As a result, different breeds of broilers may exhibit various intestinal bacterial communities and immunological status, even when grown in the same environment and fed the same diet. Considering that male broilers generally have higher growth rates than female broilers, the sex-related physiological growth may selectively influence bacterial colonization (Kers et al. 2018). Therefore, host-related biological elements (e.g. age, breed and sex) can affect gut microbial composition and activity as well as immune responses. Dietary treatments including probiotics and phytobiotics may interact with the host-related biological elements and boost or discount their impacts on gut microbial community and immune responses (Kers et al. 2018).

The present study aimed to bridge the gap between diet, host, gut microbial activity, physiology and immune responses. In order to achieve that, this study investigated the effect of age, breed, and sex of broilers (host-related factors) as well as inclusion of a probiotic or phytobiotic product (nutritional treatment) on mucosal morphology, goblet cell count, bacterial metabolites, as well as mRNA expression of the cytokines and the proteins involved in mucus production and epithelial tight junction in the ileum.

## Materials and methods

### Animals and experimental diets

A total of 2,880 one-day-old male and female broiler chicks consisting of 1,440 Ross308® and 1,440 Cobb500® were randomly allocated into 72 pens (2.25 m^2^) with a softwood shaving floor. The allocation of chicks to pens was based on breed and sex, with individuals of the same breed and sex housed together in each pen. The sex of day-old chicks was determined through vent sexing. All birds were vaccinated against Avian Infectious Bronchitis and Gumboro diseases according to the vaccination program at the hatchery and examined upon arrival (e.g., general behavior, physical appearance, and feathers). The housing system used in this study was described in Tous et al. (2022). In brief, the barn was equipped with an automatic environment control system. The light program consisted of 24 h of light for the first 2 days, followed by 18 h of light until day 7, and 14 h of light per day thereafter. The temperature program was initially set at 32–34 °C for the first 2 days, then reduced to 29–31 °C from day 3 to 7, and subsequently decreased by 3 °C per week until it reached 21 °C.

Three experimental diets including a standard wheat-soybean based diet without (CO) or with supplementation of either a probiotic (PO) or a phytobiotic (PY) product were produced and randomly assigned to birds. The trial was conducted with a 3 × 2 × 2 factorial arrangement of diet, breed and sex in a completely randomized design and consisted of 6 replicate-pens per treatment and 40 birds per pen (24 replicate-pens per diet, 36 replicate-pens per sex and 36 replicate-pens per breed). The experiment lasted 37 days. The experimental diets (starter diets for day 0–7, grower diets for day 8–21 and finisher diets for day 22–37) were formulated (Table 1) to meet or exceed recommendations of FEDNA (2018). The diets were offered in crumble form for the starter period and in 3 mm pellets later on. The probiotic product (GalliPro EPB5, Chr. Hansen, Denmark) which consists of *Bacillus subtilis* DSM32324 and DSM32325 and *B. amyloliquefaciens* DSM25840 was added into the PO diets at a dosage of 2.4 × 10^9^ CFU/kg diet. The concentration of probiotics in the diets was measured and was on average 3.7 × 10^9^ CFU/kg. The phytobiotic product (NutriPhy® White Grape 100, Chr. Hansen, Denmark) was included into the PY diets making a final concentration of 165 ppm procyanidin and 585 ppm total polyphenol in the diets. The applied dosages were according to the manufacturer recommendation.

**Table 1:** Dietary ingredients and nutrient composition of the experimental diets (as-fed basis)

Ingredients (g/kg)	Starter (0–7 days old)	Grower (8–21 days old)	Finisher (22–37 days old)
Wheat	528	612	620
Soybean meal (48% CP)	394	305	159
Soybean oil	41.6	48	0
Animal Fat (5 SYSFEED)^1^	-	-	40.1
Extruded soybean	-	-	150
Dicalcium phosphate	18.5	16.6	15
Calcium carbonate	5.3	4.8	4.4
Vitamin-mineral premix^2^	4	4	4
Sodium chloride	3.7	3.7	3.5
dl-methionine	2.7	2.3	1.9
L-lysine HCl	1.6	1.9	1.5
L-threonine	0.5	0.5	0.4
Choline chloride	0.3	0.5	0.5
Antioxidant (Noxyfeed 56P)^3^	0.2	0.2	0.2
Sodium bicarbonate	-	0.1	0.02
**Calculated nutrients and energy (g/kg, unless noted)**			
AME, kcal/kg	2900	3000	3100
Lysine	14.2	12.1	10.8
Methionine + cysteine	10.1	8.8	8.1
Threonine	9.3	7.9	7.2
Calcium	9.6	8.7	8.1
Total phosphorus	6.9	6.3	6.0
Sodium	1.6	1.6	1.6
**Analyzed nutrients (g/kg)**			
Dry matter	892	894	901
Crude protein	245	213	201
Ether extract	57	63	84
Ash	58	52	49

^1^ Product of Sysfeed SLU (Granollers, Spain, containing 1.5% myristic acid (C14:0), 18% palmitic acid (C16:0), 2% palmitoleic acid (C16:1 n-7), 14% stearic acid (C18:0), 28% oleic acid (C18:1 n-9 cis), 12% linoleic acid (C18:2 n-6 cis) and 6% α-linolenic acid (C18:3 n-3 cis).

^2^ One kg of feed contains: Vitamin A: 10 000 IU; Vitamin D3: 4 800 IU; Vitamin E: 45 mg; Vitamin K3: 3 mg; Vitamin B1: 3 mg; Vitamin B2: 9 mg; Vitamin B6: 4.5 mg: Vitamin B12: 40 µg; Folic acid: 1.8 mg; Biotin: 150 µg; Calcium pantothenate: 16.5 mg; Niacin: 65 mg; Mn (as MnSO4.H2O): 90 mg; Zn (as ZnO): 66 mg; I (as KI): 1.2 mg; Fe (as FeSO4.H2O): 54 mg; Cu (as CuSO4.5H20): 12 mg; Se (as NaSeO3): 0.18 mg; BHT: 25 mg; Calcium formiate, 5 mg; Silicicic acid, dry and precipitated, 25 mg; Calcium stearate, 25 mg; Calcium carbonate to 4 g.

^3^ Product of Itpsa (Barcelona, Spain), containing 56% of antioxidant substances (butylated hydroxytoluene + propyl gallate), 14% of citric acid and 30% of sepiolite as carrier.

### Sample collection

At day 7, 21, and 35 of age, six birds per pen were randomly selected, weighed, slaughtered and used for sample collection. The one which had the closest body weight to the averaged pen-weight was used for the present analysis (6 birds per treatment). The birds selected for the analysis were sacrificed in compliance with the ethical requirement RD 53/2013 (Spain). Following euthanasia, the birds were individually collected for ileal digesta and tissue. The digesta was collected from the distal one-third of the ileum and subsequently were frozen in liquid nitrogen and stored at -80 °C until further analysis. The distal ileal tissue was collected and used for histomorphological analyses and mRNA expression of the proteins related to epithelial barrier and inflammatory markers. For histological measurement, the tissues were fixed in 4% (vol:vol) phosphate-buffered formaldehyde immediately after slaughtering and then transferred to 70% ethanol until further analysis. For mRNA expression analysis, the entire tissues were stored in RNAlater buffer (Qiagen GmbH, Hilden, Germany) at -80 °C until further analysis.

### Histomorphological analyses

All tissue samples collected at day 7, 21, and 35 of broiler age were dehydrated, cleared with xylene and embedded with paraffin. Serial of 3 μm sections were prepared, mounted on glass slides and stained with Alcian blue-periodic acid-Schiff (AB-PAS) following manufacture’s protocol (AB-8GX, Sigma; Schiff’s reagent, Merck, Darmstadt, Germany). Ten villi and ten crypts per sample were randomly selected for morphological analysis. Villus height (*VH*) was measured from the tip to the base of the villus. The villus width (*VW*) was determined at the midpoint of the villus. Crypt depth (*CD*) was defined as its invagination depth. The villus height to crypt depth (*V/C*) was calculated from VH divided by CD. The villus surface area (*VSA*) was calculated by multiplying VH with VW. Acidic (blue), neutral (pink), mixed (purple) and total goblet cell (*GC*) were counted for each villus (GC number) and calculated as the number of GC per 100 μm of VH (GC density). All measurements were performed with an Olympus light microscope (BX 43, Olympus, Germany), which was equipped with a digital camera (DP72, Olympus, Germany). Image analysis was performed by using cellSens Standard software (version 1.14, Olympus, Germany) and ImageJ software (Rasband, W.S., ImageJ, U. S. National Institutes of Health, Bethesda, Maryland, USA).

### Metabolite analyses

Analysis of short chain fatty acids (SCFA), including acetate, propionate, i- and n-butyrate, i- and n-valerate was performed by gas chromatography on an Agilent 6890 gas chromatography system with a flame ionization detector and autosampler (Agilent Technologies, Böblingen, Germany). The separation of compounds was achieved by using the column Agilent 19,095 N-123 HP-INNOWAX polyethylene glycol (Agilent Technologies, Böblingen, Germany). D- and L‐lactate were analyzed by high‐performance liquid chromatography on an Agilent 1100 chromatograph equipped with a Phenomenex C18 (4.0 × 2.0 mm^2^) guard column followed by a Phenomenex Chirex 3126 (D)‐penicillamine column (150 × 4.6 mm^2^) and a UV detector at 253 nm. Ammonia was quantified using the Berthelot reaction assay and a photometric measurement was carried out at 620 nm. These methods were described by Goodarzi Boroojeni et al. (2014). Biogenic amines (putrescine, cadaverine, histamine, spermidine and spermine) were analyzed with reversed-phase high pressure liquid chromatography (HPLC) as described earlier (Rehman et al. 2008).

### RNA isolation and real time-quantitative PCR

Sample preparations and real-time PCR conditions have been previously described (Duangnumsawang et al. 2022). Briefly, entire tissue samples of the ileum were homogenized in buffer provided in the NucleoSpin® RNA Plus kit and RNA was isolated from the resulting tissue homogenates with the NucleoSpin® RNA clean-up according to the manufacturer’s recommendations (Macherey-Nagel GmbH & Co. KG, Düren, Germany). The mRNA quality and quantity were analyzed by a Bioanalyzer (Agilent 2100, Agilent, Waldbronn, Germany). Subsequently, reverse transcription of 100 ng of total RNA into cDNA in a final volume of 20 µL was executed using the Super Script III Reverse Transcriptase First-Strand cDNA Synthesis System (Invitrogen, Carlsbad, California). Primers used for the interleukin (*IL*)*-1β*, *IL-2*, *IL-4*, *IL-6*, *IL-8*, *IL-10*, *IL-12*, *IL-17α*, *IL-18*, Tumor necrosis factor-α (*TNF-α*), interferon γ (*IFN-γ*), transforming growth factor-beta 2 (*TGF-β2*), Mucin 2 (*MUC2*) and Claudin 5 (*CLDN5*) are presented in Table 2. The RT-qPCR was conducted with a Stratagene MX3000p (Stratagene, Amsterdam, The Netherlands). The reference mRNA level of β-actin, glycerinaldehyde-3-phosphate-dehydrogenase (*GAPDH*) and β2-microglobulin were used for normalization and times-fold expression was determined based on mean cycle threshold values of the references using the software tool REST© (Pfaffl 2002). The mRNA expression of all cytokines, *MUC2* and *CLDN5* was calculated as copy number per ng of total RNA. Then this value was divided by mean copy number of the references to obtain the expression of targeted mRNA in different treatment groups.

**Table 2:** Primer sequences used for RT-qPCR analysis

Targets^1^	Sequences of primers (5′ to 3′)	A_T_^2^	Reference
*IL-1β*	GACATCTTCGACATCAACCAG CCGCTCATCACACACGACAT	60	(Duangnumsawang et al. 2022)
*IL-2*	TCTGGGACCACTGTATGCTCT ACACCAGTGGGAAACAGTATCA	60	(Hong et al. 2006)
*IL-4*	AACATGCGTCAGCTCCTGAAT TCTGCTAGGAACTTCTCCATTGAA	60	(Avery et al. 2004)
*IL-6*	CTGCAGGACGAGATGTGCAA AGGTCTGAAAGGCGAACAGG	60	(Duangnumsawang et al. 2022)
*IL-8*	GGCTTGCTAGGGGAAATGA AGCTGACTCTGACTAGGAAACTGT	60	(Hong et al. 2006)
*IL-10*	GGAGGTTTCGGTGGAAGGAG GTTAAGCTGCCATTGAGCCG	60	(Duangnumsawang et al. 2022)
*IL-12*	AGACTCCAATGGGCAAATGA CTCTTCGGCAAATGGACAGT	60	(Hong et al. 2006)
*IL-17α*	AAGCGGTTGTGGTCCTCAT CTCCGATCCCTTATTCTCCTC	60	(Hong et al. 2006)
*IL-18*	GGAATGCGATGCCTTTTG ATTTTCCCATGCTCTTTCTCA	60	(Hong et al. 2006)
*TNF-α*	CTCGTTGGTGTGGGACGAC CGGCGGCGTATCGAAGTA	60	(Duangnumsawang et al. 2022)
*IFN-γ*	CTCCCGATGAACGACTTGAG CTGAGACTGGCTCCTTTTCC	60	(Sadeyen et al. 2004)
*TGF-β2*	TGCACTGCTATCTCCTGA ATTTTGTAAACTTCTTTGGCG	60	(Sundaresan et al. 2008)
*MUC2*	TGGCTGTGTAACTGCACCAA GTGGGTTTAGGAGGTGGCTC	60	(Duangnumsawang et al. 2022)
*CLDN5*	CATCACTTCTCCTTCGTCAGC GCACAAAGCTCTCCCAGGTC	60	(Osselaere et al. 2013)
β-actin	GAGAAATTGTGCGTGACATCA CCTGAACCTCTCATTGCCA	60	(Li et al. 2005)
*GAPDH*	GGTGGTGCTAAGCGTGTTA CCCTCCACAATGCCAA	60	(Li et al. 2005)
β2-microglobulin	AAGGAGCCGCAGGTCTAC CTTGCTCTTTGCCGTCATAC	60	(Li et al. 2005)

^1^ Three references including β-actin, *GAPDH* (glycerinaldehyde-3-phosphate-dehydrogenase) and β2-microglobulin were used as house-keeping genes. IL, interleukin; *TNF-α*, Tumor necrosis factor alpha; *IFN-γ*, interferon gamma; *TGF*-β, transforming growth factor beta; *CLDN5*, Claudin 5; and *MUC2*, Mucin 2

^2^ A_T_, annealing temperature (°C)

### Statistical analysis

Statistical analysis was conducted using SPSS 26 (SPSS Inc. Chicago, IL, United States). Data were analyzed by GLM procedure (using ANOVA) to evaluate the main factors including three ages (day 7, 21 and 35 of age), three dietary treatments (CO, PO and PY), two breeds (Ross and Cobb), and two sexes (male and female). A Four-Way ANOVA was performed to evaluate interactions between the main factors. Means were separated by the Tukey least significant difference post hoc test at *p* < 0.05 statistical level. Means and pooled standard error of the mean (SEM) were reported for all variables measured. Replicate-pen was the experimental unit for all variables measured. Figures were illustrated in GraphPad Prism 9.0.2 for Windows (GraphPad Software, San Diego, California United States).

## Results

When there was no significant interaction effect between the main factors, only the results of the main effects will be addressed. No interaction effect between age, dietary treatment, breed and sex on histomorphology of the ileum was observed (supplementary Table 1A), except for GC density (supplementary Table 1B). The effect of the main factors on histomorphology of the ileum is shown in Table 3. Age affected all the morphological variables measured. Overall, the measurement of VH, VW, and CD (expressed as µm), as well as the ratio of V/C and VSA (µm^2^) increased between 7 and 21 days of age by 64%, 28%, 51%, 11% and 109%, while only VH, CD and VSA showed a further increase (by 13%, 14% and 22%, respectively) from day 21 to 35 (*p* < 0.05). The effect of dietary treatment, breed and sex was not significant for theses morphological variables (*p* > 0.05), except for VH which was slightly higher for female birds (5.6%) compared with male ones (*p* < 0.05).

**Table 3:** The effect of age, dietary treatment, breed and sex on histomorphology in the ileum of broilers ^1^

Parameters^*^	Age (A)			Treatment (T)			Breed (B)		Sex (S)		SEM	*p*-value			
	7	21	35	CO	PO	PY	Ross	Cobb	Male	Female		A	T	B	S
**Morphology** ^2^															
VH	343 ^c^	564 ^b^	636 ^a^	520	511	516	524	507	502 ^b^	530 ^a^	10.7	< 0.001	0.994	0.124	0.034
VW	109 ^b^	139 ^a^	147 ^a^	133	129	133	131	133	132	132	2.2	< 0.001	0.767	0.845	0.871
CD	99 ^c^	149 ^b^	170 ^a^	143	139	138	141	139	138	142	2.9	< 0.001	0.746	0.409	0.423
V/C	3.5 ^b^	3.9 ^a^	3.9 ^a^	3.7	3.7	3.8	3.8	3.7	3.7	3.8	0.05	0.009	0.567	0.365	0.164
VSA	37.6 ^c^	78.4 ^b^	95.3 ^a^	71.3	68.7	72.2	71.9	69.5	69.7	71.8	2.31	< 0.001	0.818	0.374	0.541
**Goblet cell number** ^3^															
Acidic	7.5 ^b^	11.8 ^b^	34.0 ^a^	17.6	17.2	17.3	17.9	16.8	18.0	16.6	1.39	< 0.001	0.942	0.539	0.860
Mixed	75.6 ^b^	111.4 ^a^	115.3 ^a^	100.5	97.7	103.5	103.7	97.3	94.2 ^b^	107.2 ^a^	2.63	< 0.001	0.618	0.135	0.004
Total	83.1 ^c^	123.2 ^b^	149.3 ^a^	118.1	114.8	120.7	121.6 ^a^	114.1 ^b^	112.2 ^b^	123.8 ^a^	2.67	< 0.001	0.515	0.026	0.001
**Goblet cell density** ^4^															
Acidic	2.1 ^b^	2.2 ^b^	5.4 ^a^	3.3	3.1	3.2	3.2	3.2	3.3	3.1	0.22	< 0.001	0.967	0.926	0.820
Mixed	22.3 ^a^	19.8 ^b^	18.1 ^b^	19.5	20.1	20.5	19.7	20.4	19.3 ^b^	20.8 ^a^	0.37	< 0.001	0.510	0.363	0.039
Total	24.5 ^a^	21.9 ^b^	23.4 ^ab^	22.8	23.2	23.8	22.9	23.6	22.6 ^b^	23.9 ^a^	0.30	0.002	0.362	0.289	0.017

^1^ The trial was conducted with a 3 × 2 × 2 factorial arrangement of diet, breed and sex in a completely randomized design and consisted of 6 replicate-pens per treatment and 40 birds per pen. Data were subjected to ANOVA using GLM procedure to evaluate age, diet, breed and sex.

^2^ Villus height (VH), villus width (VW), and crypt depth (CD) are measured in µm, V/C ratio was calculated by dividing villus height with crypt depth, villus epithelial surface area (VSA) was calculated by the multiplication of villus height and villus width, expressed as 10^3^ µm^2^.

^3^ The average number of goblet cells per villus. Acidic represents the cells that are positive to Alcian blue dye. Mixed represents the cells that are positive to both Alcian blue and PAS dye. Total represents the sum of acidic and mixed goblet cells.

^4^ The average number of goblet cells per 100 μm villus height. Acidic represents the cells that are positive to Alcian blue dye. Mixed represents the cells that are positive to both Alcian blue and PAS dye. Total represents the sum of acidic and mixed goblet cells.

^a,b,c^ Means within a row of each main factors lacking a common superscript differ (*p <* 0.05).

^*^ CO, Control; PO, Probiotic product; PY, Phytobiotic product.

**Fig. 1:**
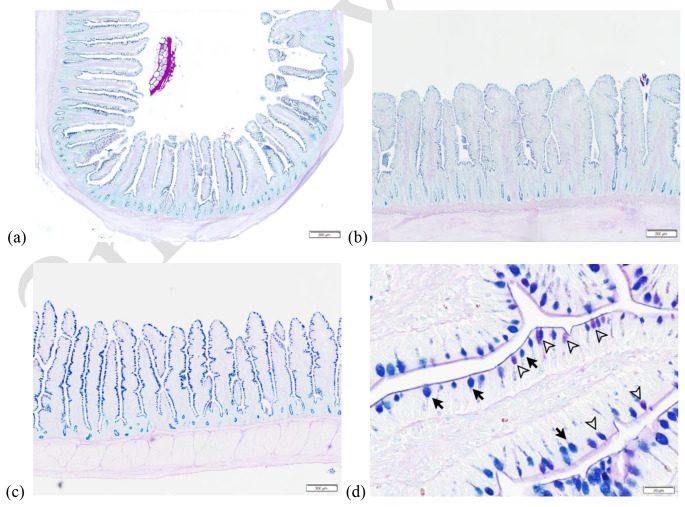
Alcian blue-periodic acid-Schiff stain on the ileal tissue. Low magnification (10x, a-c) shows the positive stained mucins (blue-purple color) in the goblet cells lining along the villus and crypt of the ileum at day 7 (**a**), day 21 (**b**), and day 35 (**c**). High magnification (100x, **d**) of the villus part of the ileum shows the goblet cells containing blue-stained mucins (acidic goblet cells, solid black arrow) and purple-stained mucins (mixed goblet cells, open arrowhead). The magenta-stained mucins (neutral goblet cells) were not observed in this sample.

Along the villi, approximately 79–88% of the detected GC seemed to be mixed type, while the remaining GC (12–21%) were mainly acidic type (Fig. 1). Neutral type of GC was not present in most of the samples and when present, their number was negligible. The number of acidic, mixed and total GC (per villi) was affected by age (*p* < 0.05). The number of mixed and total GC increased from day 7 and 21 (*p* < 0.05) by approximately 47% and 48%, respectively, however, the 57% increase in number of acidic GC from day 7 to 21 was not statistically significant (*p* > 0.05). The numbers of acidic and total GC further increased from day 21 to 35 (*p* < 0.05) by around 188% and 21%, respectively, while the number of mixed GC remained stable. The density of GC (per 100 μm VH) was also affected by age (*p* < 0.05). The density of acidic GC was similar at day 7 and 21 of age (*p* > 0.05), while it increased by 145% from day 21 to 35 (*p* < 0.05). In contrast, the density of mixed GC and total GC decreased by 11.2% and 10.6% from day 7 to 21 (*p* < 0.05) and remained constant from day 21 to 35 of age (*p* > 0.05). Breed only affected the number of total GC (*p* < 0.05) with Ross showing 6.2% greater total GC number than Cobb. Sex had an impact on both GC number and density in the ileum and females showed slightly higher number (13.8% and 10.3%, respectively) and density (7.8% and 5.8%, respectively) of mixed and total GC compared with males (*p* < 0.05). However, no differences were observed for acidic GC number and density of males and females (*p* > 0.05). No influence of dietary treatments was found for the GC measurements (*p* > 0.05). The only significant interaction was between age and sex for total GC density (*p* < 0.05, supplementary Table 1B) and females showed higher total GC density at day 35 compared with males at the same age and both females and males at day 21 (*p* < 0.05). Females also showed greater total GC density at day 7 compared with females at day 21 and males at day 35 (*p* < 0.05).

**Fig. 2:**
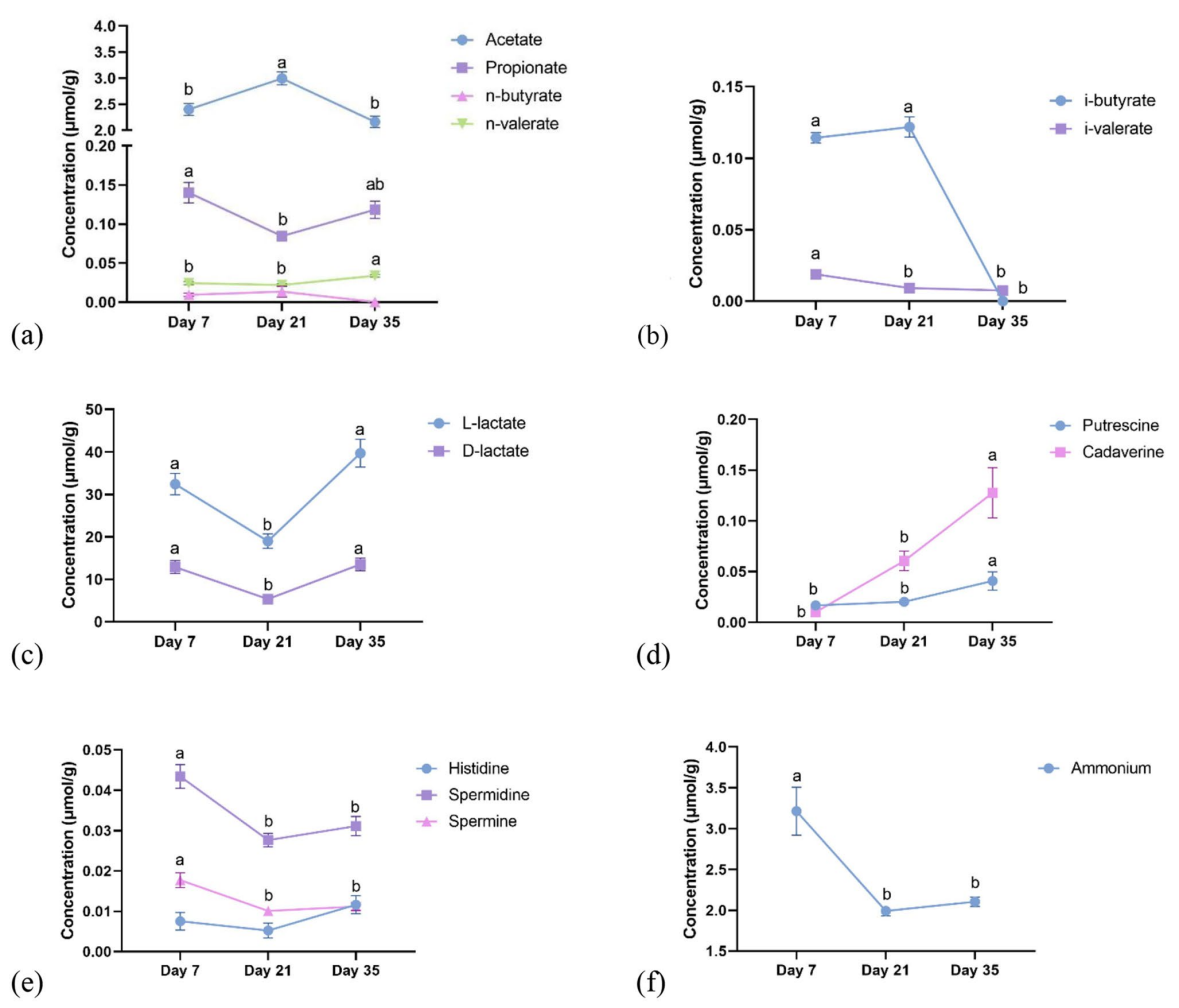
(**a-f**) The effect of age (day 7, 21, and 35 of age) on metabolite concentration (µmol/g of fresh sample) in the ileum of broilers. The trial was conducted with a 3 × 2 × 2 factorial arrangement of diet, breed and sex in a completely randomized design and consisted of 6 replicate-pens per treatment and 40 birds per pen. Data were subjected to ANOVA using GLM procedure to evaluate age, diet, breed and sex. All the data was presented in supplementary Table 2A and 2B.

**Fig. 3:**
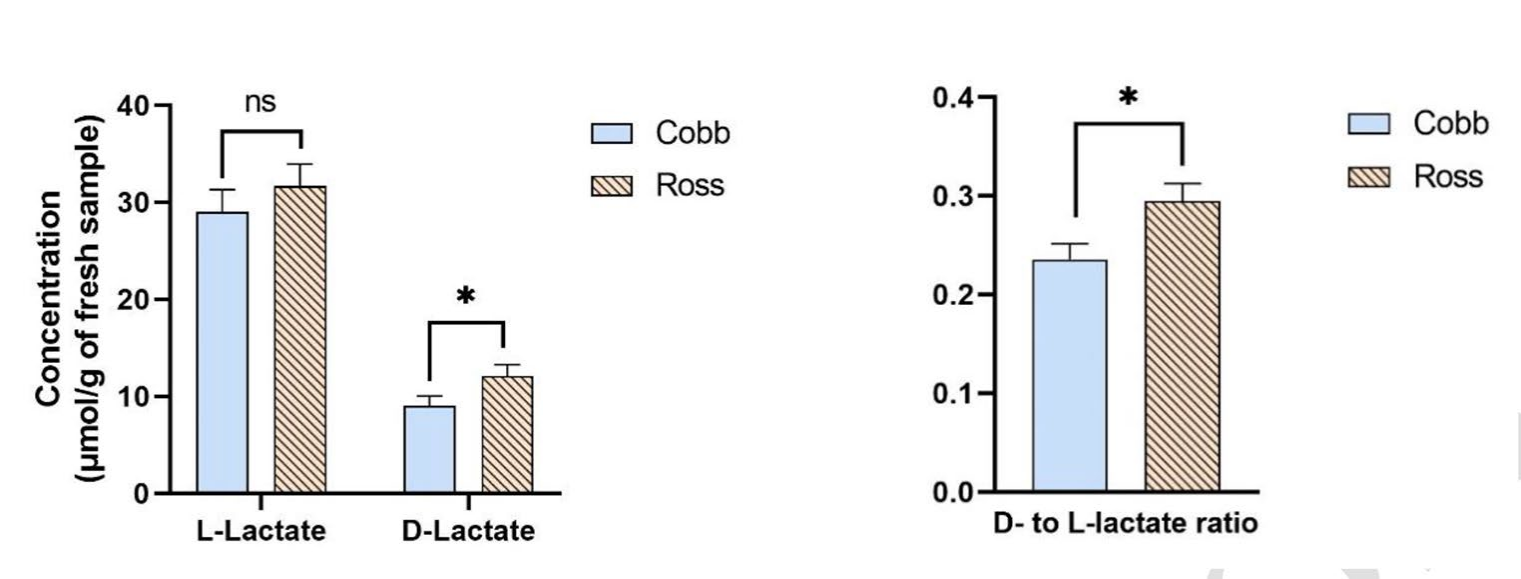
(**a-e**) The effect of breed on L- and D-lactate concentration (µmol/g of fresh sample) and its ratio in the ileum of broilers. The trial was conducted with a 3 × 2 × 2 factorial arrangement of diet, breed and sex in a completely randomized design and consisted of 6 replicate-pens per treatment and 40 birds per pen. Data were subjected to ANOVA using GLM procedure to evaluate age, diet, breed and sex. All the data were presented in supplementary Table 2A and 2B. *, significant difference (*p* < 0.05); ns, no significant difference (*p* > 0.05)

The effects of the main factors on metabolite concentration (µmol/g of fresh sample) of the ileum are presented in Figs. 2 and 3 and supplementary Table 2A and 2B. The significant interaction effects on bacterial metabolites are shown in supplementary Table 2C and 2D. The main effect of dietary treatment, breed and sex had no impact on ileal metabolites concentrations (*p >* 0.05), except for D-lactate and D- to L-lactate ratio which was higher for Ross than Cobb (*p <* 0.05, Fig. 3). Age was the only main factor that altered concentration of all metabolites measured in the ileum (*p <* 0.05, Fig. 2), except for n-butyrate and histamine. Concentration of acetate accounted for approximately 90% of the total SCFA in the ileum, followed by propionate (4%), i-butyrate (3%) and n-valerate (1%). Concentration of n-butyrate and i-valerate in ileal digesta was almost negligible (less than 0.4% of total SCFA). Concentration of acetate increased by 24.6% from day 7 to 21 and then decreased by 27.8% at day 35 (*p <* 0.05, Fig. 2a). Propionate concentration decreased by 42.9% from day 7 to 21 and then increased by 50.0% thereafter (*p <* 0.05, Fig. 2a). Concentration of n-valerate was higher in 35 days old broilers compared with the younger ones (*p <* 0.05), while n-butyrate was not affected by age (*p >* 0.05, Fig. 2a). Both i-butyrate and i-valerate concentrations decreased with age (*p <* 0.05, Fig. 2b) and were almost absent at day 35. Concentration of D- and L‐lactate, total lactate and their ratio (D‐ to L‐) was lowest at day 21, while those variables were not different between day 7 and 35 (*p <* 0.05, Fig. 2c). Concentration of D‐, L‐ and total lactate concentration in the ileum decreased by 58%, 41%, and 46% between day 7 and 21, and increased by 152%, 109%, and 119% between day 21 and 35 (*p <* 0.05). Concentrations of all biogenic amines were also influenced by age (*p <* 0.05), except for histamine. Putrescine and cadaverine concentration were not different between day 7 and 21 (*p* > 0.05) but, from day 21 to 35, both metabolites increased their concentration by approximately 102% and 117%, respectively (*p <* 0.05, Fig. 2d). In contrast, spermidine and spermine concentration decreased by 25% and 50%, respectively from day 7 to 21 (*p <* 0.05, Fig. 2e) and remained stable after that. Ammonium concentration reduced by 38% from day 7 to 21 (*p <* 0.05, Fig. 2f) and remained stable thereafter. An interaction between breed and sex was observed for spermidine (supplementary Table 2C). The concentration of spermidine was lower in female-Ross broilers compared with male-Ross broilers (*p <* 0.05), but both male- and female-Ross broilers showed no difference in spermidine concentration compared with Cobb broilers, regardless of sex (*p >* 0.05). An interaction between age and breed was detected for ammonium, with 7 days old Ross broilers having the highest concentration of ileal ammonium, while other groups were not different from each other (supplementary Table 2D).

**Fig. 4:**
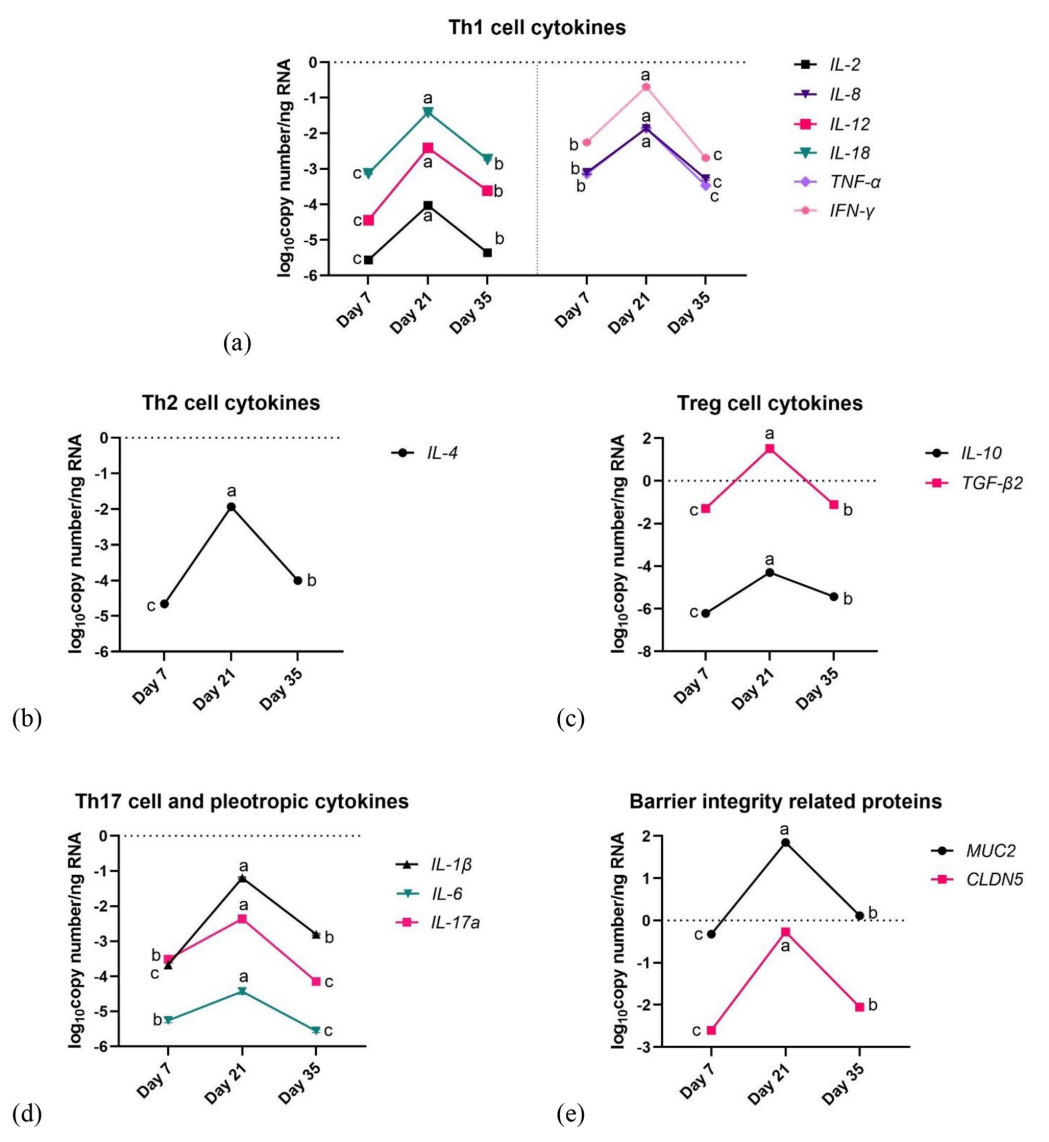
(**a-e**) The effect of age on mRNA expression in the ileum of broilers. The mRNA expression represents as log_10_ copy number per ng of RNA (calculated by dividing the copy number of targeted mRNA with the copy number of the housekeeping genes, converting values to the copy number per total RNA, and then transformed to log_10_ scale). The trial was conducted with a 3 × 2 × 2 factorial arrangement of diet, breed and sex in a completely randomized design and consisted of 6 replicate-pens per treatment and 40 birds per pen. Data were subjected to ANOVA using GLM procedure to evaluate age, diet, breed and sex. All data were presented in supplementary Table 3A and 3B. ^a, b, c^ Means with different superscripts in each variable differ significantly (*p* < 0.05). *IL*, interleukin; *TNF-α*, Tumor necrosis factor alpha; *IFN-γ*, interferon gamma; *TGF-β2*, transforming growth factor beta 2; *CLDN5*, Claudin 5; *MUC2*, Mucin 2

**Fig. 5:**
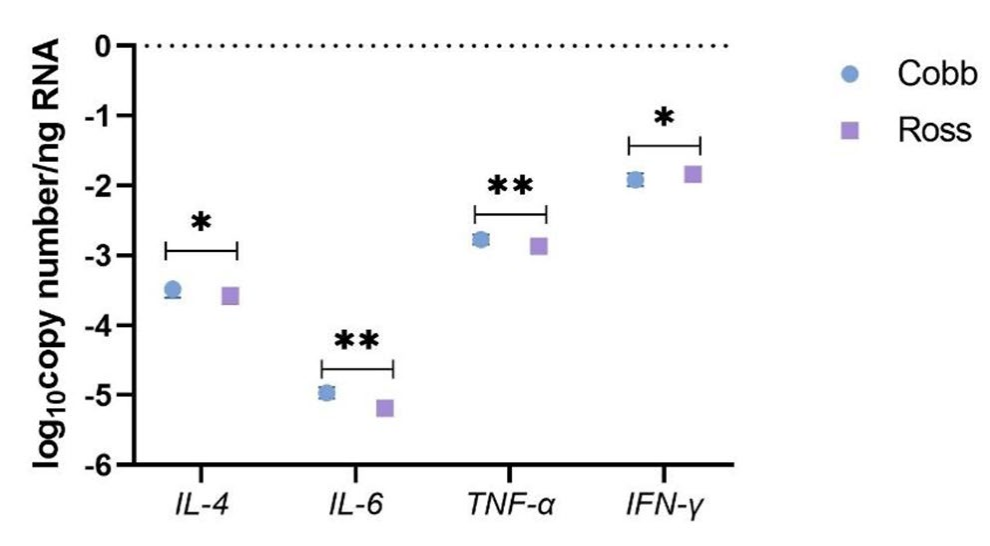
The effect of breed on mRNA expression in the ileum of broilers. The mRNA expression represents as log_10_ copy number per ng of RNA (calculated by dividing the copy number of targeted mRNA with the copy number of the housekeeping genes, converting values to the copy number per total RNA, and then transformed to log_10_ scale). The trial was conducted with a 3 × 2 × 2 factorial arrangement of diet, breed and sex in a completely randomized design and consisted of 6 replicate-pens per treatment and 40 birds per pen. Data were subjected to ANOVA using GLM procedure to evaluate age, diet, breed and sex. All data were presented in supplementary Table 3A and 3B. ^*, **^ Means in each variable differ significantly (^*^, *p* < 0.05; ^**^, *p* < 0.01). *IL*, interleukin; *TNF-α*, Tumor necrosis factor alpha; *IFN-γ*, interferon gamma

The main impacts of age, dietary treatment, breed, and sex on mRNA expression (log_10_ copy number per ng of RNA) of cytokines and the proteins related to epithelial barrier integrity of the ileum are shown in Figs. 4 and 5, and supplementary Table 3A and 3B. In addition, the significant interaction effect is shown in supplementary Table 3C. Age was the main factor that altered investigated mRNA expression (*p <* 0.05), while no impact of dietary treatment, breed and sex on the variables was observed (*p >* 0.05), except for impacts of breed on *IL-4*, *IL-6*, *TNF-α* and *IFN-γ* (*p <* 0.05). Overall, all mRNA expression of cytokines (*IL-1β*, *IL-2*, *IL-4*, *IL-6*, *IL-8*, *IL-10*, *IL-12*, *IL-17α* and *IL-18* as well as *IFN-γ* and *TGF-β2*) and epithelial barrier related proteins (*MUC2* and *CLDN5*) increased from day 7 to 21 where they reached the peak, and then decreased at 35 days of age (*p <* 0.05). Among the cytokines investigated, *IL-4* and *TGF-β2* showed a considerable change in their expression during 35 days of life; both were upregulated (537- and 631-fold) from day 7 to 21 and downregulated (117- and 417-fold) from day 21 to 35 (*p <* 0.05, Fig. 4b and c). There was an mRNA upregulation of *IL-1β* and *IL-12* by 295- and 107- fold between day 7 and 21, while a lesser degree of upregulation (between 7- and 81-fold) was found for the remaining cytokines, with the following order *IL-10* > *IL-18* > *IFN-γ* > *IL-2* > *TNF-α* > *IL-8* > *IL-17α* > *IL-6* (*p <* 0.05, Fig. 4a-d). On the other hand, mRNA expression of *IFN-γ* was downregulated by 100-fold between day 21 and 35, while a lesser degree of downregulation (between 13- and 61-fold) was found for *IL-17* > *TNF-α* > *IL-1β* > *IL-8* > *IL-2* > *IL-18* > *IL-12* > *IL-10* > *IL-6* in the following order (*p <* 0.05, Fig. 4a-d). The mRNA expression of barrier integrity related proteins including *MUC2* and *CLDN5* was upregulated by 148- and 214-fold from day 7 to 21 and downregulated by 55- and 60-fold from day 21 to 35 (*p <* 0.05, Fig. 4e).

For Cobb, mRNA expression of *IL-4*, *IL-6* and *TNF-α* was higher than Ross, while *IFN-γ* was higher for in Ross compared with Cobb (*p <* 0.05, Fig. 5). The interaction between age and breed had an impact on *IFN-γ* (*p <* 0.05, supplementary Table 3C). The expression of *IFN-γ* was highest in the ileum of Ross and Cobb at day 21 and was lowest in Ross at day 35 (*p <* 0.05). However, at day 7, *IFN-γ* expression was similar for Ross and Cobb but it was higher than Cobb at day 35 (*p <* 0.05).

## Discussion

Before discussing the present findings, it is worth mentioning that the growth performance data of broilers used in the present study as well as the data on their caecal histomorphology, GC density, bacterial metabolites, and mRNA expression of cytokines and proteins related to intestinal integrity have been already published by Tous et al. (2022) and Duangnumsawang et al. (2022).

Over the past decade, it has been revealed that gut microbiota and their metabolites play a vital role in gut health of broilers and alter development and functionality of the gut and its immune system (Tang et al. 2020). Host-related factors including age, breed, and sex have been shown to affect intestinal microbial community and immune responses (Torok et al. 2013; Richards-Rios et al. 2020). Genes are known to contribute to variation in physiological traits. Genotypic variations of broiler breeds and sexes may influence their gut physiology which creates a specific environment for microbial colonization. Differences in microbial composition and activity could result in distinct immune traits in the gut (Kers et al. 2018). In the present study, female broilers showed longer villi and higher number and density of mixed and total GC compared with male broilers, while their VW, CD, V/C, and VSA were similar. Studies reviewed by Heak et al. (2017) suggested that greater VH may be associated with increased surface area for nutrient absorption, while an increased number of GC may be linked to greater production of intestinal mucin resulting in higher endogenous loss (e.g., energy and protein) to the birds (Duangnumsawang et al. 2021). As a result, differences in these morphological variables may have an impact on growth performance between male and female broilers. Male birds showed better growth performance in terms of body weight gain, feed intake, and feed conversion ratio compared with females (Tous et al. 2022). However, it seems difficult to draw a clear conclusion regarding significance of the observed differences in gut morphology of male and female broiler chickens for their growth performance. In terms of the differences between breeds, Ross chickens had a higher total GC number, D-lactate concentration and D‐ to L‐lactate ratio than Cobb broilers. In addition, Ross showed an upregulation of *IFN-γ*, while Cobb had a higher expression of *IL-4*, *IL-6*, and *TNF-α*. However, no significant differences between breeds were found for other variables measured (*p* > 0.05). A similar pattern for cytokine expression has been also reported in the caecum of the current broilers (the same trial, focusing on the caecum) by Duangnumsawang et al. (2022). Alterations in bacterial metabolites such as lactate (which is important energy source for the host) and differences in cytokine expression between breeds have been reported to affect immune responses and growth performance of the birds (Gadde et al. 2017; Lee et al. 2018). Differences in growth performance of these current birds (the same trial focusing on growth performance) were also observed, with Cobb having higher body weight gain, and feed intake, as well as lower feed conversion ratio compared with Ross (Tous et al. 2022). Therefore, differences in growth performance between Ross and Cobb may be attributed to variations in bacterial metabolite concentration and cytokine expression in the gut but the mode of actions behind it is not clear. Nevertheless, a nearly identical gut microbial activity and immune responses for both breeds and sexes in the present study could be attributed to the absence of harmful stimuli which are reportedly responsible for disrupting intestinal microbial populations and resulting in microbial dysbiosis, and induce gut immune responses in order to protect the gut from potential injuries (Mabelebele et al. 2017; Paraskeuas and Mountzouris 2019; (Wang et al. 2021b).

Modifications in gut microbiota of broilers induced by probiotics and phytobiotics have been demonstrated to be advantageous for gut homeostasis with promoting proliferation and metabolic activities of beneficial bacteria and suppressing those of pathogenic species (Krysiak et al. 2021). These modifications have been linked to improved growth performance in broilers (Lee et al. 2015). In this study, adding multi-strain *Bacillus* based probiotic or a procyanidin-rich phytobiotic to broiler diets did not show any impact on bacterial metabolic activity, morphology and mRNA expression of cytokines and proteins associated with mucus production and epithelial integrity in the ileum. In addition, the applied probiotic or phytobiotic product did not affect growth performance of the current birds (Tous et al. 2022). Broilers fed diets with *B. subtilis* showed an unchanged bacterial diversity (e.g. Shannon index) in the caecum (by using 16s rRNA analysis) compared with those fed control diet (Lin et al. 2017; Jacquier et al. 2019). However, these studies displayed that *Bacillus* spp. altered bacterial genera and their functional activities related to activation of immune responses in the ileum of broilers. Several studies showed that modification of gut microbiota by dietary *Bacillus* spp. improved gut barrier integrity and activated immune response in the ileum of broilers, as shown by an upregulation of tight junction proteins (e.g. occludin, *ZO-1* and *JAM-2*) and mucin (e.g. *MUC2*) as well as cytokines (e.g. *IL-1β*, *IL-12*, *IFN-γ* and *IL-10*) (Rajput et al. 2013; Lee et al. 2015; Bilal et al. 2021). *Bacillus* based probiotics used in previous studies showed positive effects on gut barrier integrity and modulate host immune system, but the extent of these effects varied from strain to strain and also seemed to be dependent on dietary inclusion level, diets composition, environmental condition and age of animals (Yaqoob et al. 2022). Grape extract was found to affect bacterial metabolic activity in the gut through modulating phenolic metabolism of bacteria (Chamorro et al. 2019). An abundant source of polyphenolic compounds in grapes, mainly procyanidins, have been linked to reduced oxidative stress and intestinal inflammation in broilers (Chamorro et al. 2019). In the current study, beneficial impacts of grape extract on ileal bacterial activity, morphology, and immune responses of broilers have been scarce. Previous reports showed that polyphenol rich grape extracts could suppress pro-inflammatory cytokines (e.g. *IL-1β*) in the gut of broilers (Cao et al. 2020), while they stimulated anti-inflammatory cytokines including *IL-10* and *TGF-β1* in Caco-2 human colon cells (Nallathambi et al. 2020) and caecum of broilers (Duangnumsawang et al. 2022). Increasing grape procyanidin level in broiler diets has been shown to reduce concentration of sialic acid in ileal digesta, which may reflect the direct or indirect (through microbial alterations) effect of procyanidins in modifying mucin composition (Chamorro et al. 2019). However, adding procyanidin rich phytobiotic to broiler diets in the present study did not alter GC count and expression of *MUC2* (related to mucin production) in the ileum, which is in line with our previous study evaluating the same variables in the caecum of broilers (Duangnumsawang et al. 2022). The observed inconsistency in the outcome of different studies testing grape extracts could be because of environmental condition, experimental diet composition, molecular structure (e.g. degree of polymerization of procyanidins) and concentration of the active substances in the final diets (González-Quilen et al. 2020), as well as host-related factors, for instance, age, breed, and sex, which differed between trials and could have impacted gut microbiota development (Kers et al. 2018).

In the current study, ileal microbial metabolites, gut morphology and immune traits changed during the growth period. During co-development of the host and gut microbiota, products of bacterial metabolic activity including SCFA and lactate, could be the main factors modulating the host immune system (Yang et al. 2017b). At 35 days of age, lactate presented the highest concentration in the ileum with an average of 40.9 ± 2.29 mol/g, followed by acetate with an average of 2.5 ± 0.07 mol/g of fresh digesta. However, the age-related changes in concentration of acetate and lactate were in opposite directions; acetate concentration were highest on day 21, while lactate concentration was at its lowest point. In another study, the same pattern was observed for SCFA (increase) and lactate (decrease) concentration in the caecum of broilers after 2 weeks of age and it was attributed to the direct effect of lactate-utilizing bacteria or indirect effect of bacterial groups that playing a role in metabolic cross-feeding of fermentation products (Meimandipour et al. 2011). In this study, concentration of propionate, i- and n-valerate, and i-butyrate were present at low levels (up to 4% of total SCFA), while n-butyrate was nearly undetectable in the ileum, which seems to be in line with previous reports (Goodarzi Boroojeni et al. 2014; Liao et al. 2020). Biogenic amines, primarily putrescine and cadaverine, were found at a relatively low concentration during the first 21 days of life in this study, while their concentration increased by around 2-fold at day 35. Another study also found increased cadaverine levels (around 2-fold) in the ileum of older broilers (day 41) compared with younger ones (day 20), but putrescine levels slightly decreased as broilers aged (Tiihonen et al. 2010). The derivatives of putrescine including spermine and spermidine were present at low level (up to 0.05 µmol/g fresh digesta) in the ileum and their concentration was higher in young birds (day 7) compared with the older ones (day 21 and 35). Biogenic amines, especially putrescine, spermine and spermidine, have been found to enhance homeostasis of the intestinal mucosa and increase the rates of epithelial cell division and apoptosis through modulating the expression of various growth-related genes (Timmons et al. 2013). Thus, increasing the concentration of biogenic amines, especially putrescine with age in the present study may be associated with enterocytes and GC proliferation, altering villus and crypt structure as well as mucus production. This could be supported by previous study showing that putrescine *in ovo* injection enhanced cell proliferation as shown by increased VH and GC number in the ileum of broilers (Goes et al. 2021). In this study, ammonium concentration decreased and remained low after the first week of age. The putrefactive metabolites including branched chain fatty acids, biogenic amines and ammonium are products of protein fermentation, while SCFA and lactate are mainly derived from saccharolytic (carbohydrate) fermentation (Qaisrani et al. 2015). Thus, the concentration of these metabolites could be altered by availability of nutrients for the gut microbiota as well as the number and metabolic activity of protein- and carbohydrate-fermenting microorganisms. Using 16s rRNA sequencing has been shown that the relative abundance in bacterial composition and genome (gene based clone libraries) in the ileum and caecum of broilers were concurrently changed with age, during the first 7 weeks of life (Lu et al. 2003). Therefore, age-related changes in the gut bacterial metabolites may reflect alterations in microbial composition and metabolism which could be caused by different factors such as feeding transition (e.g. feed form, structure, quality and composition), nutrient digestibility of feed, environmental factors (e.g. microbial load and hygiene status) and stress (e.g. environmental and physiological stresses). However, most metabolites, such as SCFA, are quickly absorbed by intestinal cells or transformed into other types of metabolites by gut bacteria (Gomez-Osorio et al. 2021). Thus, it should be noted that measuring concentration of bacterial metabolites in fresh digesta (per weight of digesta) provides only a snapshot of bacterial activity at that particular time and may not accurately represent the actual amount of metabolites produced over time.

The interaction between the intestinal immune system and commensal microbiota in chickens begins at hatching and the host immune system simultaneously responds to changes in the luminal environment as broilers grow. Cytokines act as intercellular immunological messengers promoting intestinal mucosal homeostasis, and they can also be significant drivers of intestinal inflammation and damage (Siddiqui et al. 2020). In general, the investigated cytokines in the present study were selected according to their immune regulatory function and production by T helper (Th) cells (Lee et al. 2019): Th1 cytokines (*IL-2*, *IL-8*, *IL-12*, *IL-18*, *TNF-α*, and *IFN-γ*), Th2 cytokines (*IL-4*), Th17 (*IL-17α*) and regulatory T (Treg) cytokines (*IL-10* and *TGF-β2*) as well as pleiotropic cytokines (IL-1β and *IL-6*). As reviewed by Rescigno and Di Sabatino (2009), Th2 cells are primarily related to the secretion of B cell growth factors including *IL-4*, while Th1 cells are inflammatory cells that direct immune reactions against intracellular pathogens and Th17 cells play a critical role in host defense against a variety of bacteria and fungi. In contrast, Treg cells suppress the functions of effector T cells and are essential to counteract inflammatory responses. The activation of multiple cell types by *IL-1β* and *IL-6* was previously reported; *IL-1β* is a pro-inflammatory cytokine that stimulates Th1, Th2 and Th17 cell proliferation (Muñoz-Wolf and Lavelle 2018) and *IL-6* has both pro- and anti-inflammatory actions that activates Th17 and inhibits Treg cell proliferation (Murakami et al. 2019). In the current study, mRNA expression of all the cytokines was highest at 3 weeks of age and then decreased. In another study, expression of *TGF-β1* and *IFN-γ* in the ileum of broilers was upregulated form day 20 to 27 and then downregulated at day 34, which was associated to an increase in T and B cell proliferation activity (Song et al. 2021). In general, epithelial cells can recognize luminal antigen and transmit this information to the immune cells in the lamina propria to secrete cytokines and restore the balance in the intestine (Mahapatro et al. 2021). During the first week after hatching, antigens from diet and environment construct an immune response in the gut of broilers via recruiting granulocyte and T-lymphocyte and generating cytokines, which could trigger immunological adaptation to luminal antigens and microbiota (Van Immerseel et al. 2002; Bar-Shira et al. 2003; Crhanova et al. 2011). When immunological stimulations (dietary and environmental stimuli) in the lumen reduce, the restoration of immunological balance can take place (Broom and Kogut 2018). An immune stabilization process following a shift of bacterial composition in the gut was suggested to be a mechanism that prevent the body from entering a state of excessive immune activity and to maintain the body’s immune balance (Song et al. 2021). The temporary upregulation of all cytokines in the present study could be indicative of an overall immunological response to the physiological changes, microbial establishment/maturation and environmental stress during growth. Downregulation of all cytokines after day 21 may imply adaptation of the gut immune system to luminal antigens and microbiota after 3 weeks, leading to a lesser degree of immune stimulation in the gut. In this study, age-related changes in expression of the pro-and anti-inflammatory cytokines followed the same pattern. During the activation of pro-inflammatory pathway, the presence of anti-inflammatory cytokines may play a role in negative feedback mechanism of the inflammatory activity (Park et al. 2014). In this study, the observed age-related fluctuation of lactate and SCFA which are the main bacterial metabolites in the ileum, seemed to trigger both pro- and anti-inflammatory cytokines, which could be advantageous for immunological maturation and adaptation. Variations in ileal cytokine expression and acetate concentration appeared to be parallel and aligned, while variations in cytokines and lactate concentration were parallel but pointing in opposite directions. It has been shown that microbial metabolites such as SCFA and lactate regulate T cells differentiation and cytokine secretion (Park et al. 2014; Manoharan et al. 2021). In vitro addition of acetate, propionate, and butyrate promoted the differentiation of naïve CD4^+^ T cells to effector (Th1 and Th17) and Treg cells, resulting in an upregulation of cytokines e.g. *IL-10*, *IFN-γ*, and *IL-17* (Park et al. 2014). It was also found that in vivo regulation of the host immune system by SCFA has been attributed to the direct effect of SCFA on the immune cells or their indirect impact through the cellular signals of the intestinal epithelial cells (Park et al. 2014). Lactate could also modulate the cellular signaling of immune cells such as dendritic cells and macrophages and regulated the development of Treg/Th1/Th17 cells, resulting in an induction of immune regulatory factors and inhibition of pro-inflammatory cytokines (Ranganathan et al. 2018).

*MUC2* is a major constituent of mucins, forming a net-like structure of the intestinal mucus layer (Zhang and Wu 2020). In this study, expression of *MUC2* was upregulated during the first 21 days of age and then downregulated until day 35. A previous study showed an increased *MUC2* expression in the ileum during the first week of life and then become steady until day 14 of age (Proszkowiec-Weglarz et al. 2020). However, Zhang et al. (2015) reported a steady expression of *MUC2* in the ileum and caecum of broilers after hatching until 3 weeks of age. The pattern of *MUC2* expression could be influenced by bacterial colonization and subsequent host response that increases mucin secretion to limit the epithelial contact with intestinal bacteria (Zhang et al. 2015). The claudin family is a key component that forms epithelial tight junctions that regulate paracellular permeability, epithelial polarization, and conservation of transepithelial resistance, as well as the selective passage of molecules and ions in the chicken intestine (Turner 2009; von Buchholz et al. 2021). *CLDN5* is the main barrier-forming claudins between adjacent epithelial cells in the gut of chicken which involves in paracellular permeability and intestinal homeostasis (Ozden et al. 2010). The immunostaining of *CLDN5* has been shown to be stronger in the crypt and lower villus regions of the small intestine of newly hatched broilers compared with other *CLDN* family such as *CLDN3* (Ozden et al. 2010). Therefore, expression of *CLDN5* in ileal mucosa may be a good marker for evaluating tight junction in the ileum. Like *MUC2* and other cytokines, *CLDN5* expression reached a peak at day 21 in this study. In contrast to this study, expression of tight junction proteins including *CLDN1* and *CLDN5* in the jejunum and ileum of broilers decreased after hatch and became stable during the first 2 weeks of life. It has been discussed that expression of tight junction proteins could be a result of a compensatory mechanism responding to alterations in microbial composition and restoring intestinal permeability (Proszkowiec-Weglarz et al. 2020). Similarities in the expression patterns of *MUC2*, *CLDN5* and all the investigated cytokines may imply that alterations in immune system (e.g. cytokines) may subsequently influence mucus production and epithelial integrity of the gut. This speculation is supported by Mahapatro et al. (2021) study which demonstrated the regulatory mechanisms of pro-inflammatory cytokines (e.g. *IL-4*, *IL-18*, *IFN-γ* and *TNF*) that induced the differentiation of progenitor cells to cells of the secretory lineage such as GC and increased mucus production, thereby restoring the intestinal epithelial barrier.

The crypt-villus morphology in the ileum provides the environment for digestion and absorption, while its structure could be simultaneously affected by commensal or pathogenic microorganisms residing in the gut. In this study, all morphological variables including VH, VW, CD, V/C and VSA increased with age, with VH and VSA showing 85% and 153% increase from day 7 to 35. Longer intestinal villi are associated with an increase in the absorptive surface of the intestines which support increase in nutrients requirement during broiler growth (Awad et al. 2009). Bacterial metabolites such as SCFA and lactate are known to affect villus and crypt morphology (Lee et al. 2018). As a source of energy, butyrate plays a vital role in promoting intestinal development and maintaining the integrity of the intestinal epithelial cells (Zou et al., 2019). Acetate has been shown to alter intestinal cell apoptosis and mucus production (Liu et al., 2017). Propionate is also a potent fatty acid that modulate intestinal cell activity including differentiation and apoptosis (Hosseini et al., 2011). Lactate possesses diverse metabolic and regulatory properties, such as being an energy source and a signaling molecule for intestinal stem cell and goblet cell regeneration (Lee et al. 2018). Besides these main metabolites, as mentioned earlier, some biogenic amines also alter regeneration of the epithelial cells, while high concentration of ammonia may cause cell damage (Rehman et al. 2007). Therefore, age-related alterations in the investigated metabolites in this study may influence cell differentiation and proliferation in the ileum.

In the present study, the number of total GC (per villus) increased from day 7 to 35 of age, while the density of total GC decreased during the first 3 weeks of age and then it became stable. Other studies have also shown an increase in GC number per villus in the ileum of broilers during 3–5 weeks of age (Sikandar et al. 2017; Thiam et al. 2021). In accordance with the present data, Duangnumsawang et al. (2021) demonstrated that the GC density in the ileum is relatively high during the first week of age, but it tends to decrease afterward until the third week of life and then becomes stable between the third and fifth week of age. Mucin-secreting GC are the first line of defense in the mucosa and mucins secreted by GC can protect epithelial cells from pathogens, chemical and mechanical damages. Therefore, mucin-secreting GC develop and mature after hatch as a response to external stimuli including intestinal microbiota, dietary factors and antigens from diet and environment (Duangnumsawang et al. 2021). It has been also reported that changes in GC number of the gut could be due to biological mechanisms such as cell proliferation and apoptosis regulated by direct and/or interaction effect of gut microbiota (dysbiosis and symbiosis) and host immune response (Deplancke and Gaskins 2001). The observed pattern for age-related changes in GC density in the current study, might be also caused by immunological adaptation of the gut immune system to the luminal substances (e.g. feed, microbiota, antigens, etc.).

Mucins are the major components of the intestinal mucus layer and can be classified into neutral and acidic subtypes based on their net molecular charge. Acidic type expresses a net negative charge and neutral type exhibits a net neutral charge of the mucin molecule (Derrien et al. 2010). The distinct pattern of mucins in the gut may reflect differential host responsiveness to specific bacterial communities or metabolites (Deplancke and Gaskins 2001). In this study, the majority of GC population was presented as a mixed type (containing relatively similar proportion of acidic and neutral mucins) and the remaining GC can be categorized as acidic type, suggesting that the proportion of secreted acidic mucins in the mucus layer of the ileum may be greater than neutral mucins. High prevalence of acidic mucins was also reported in villi of the duodenum, jejunum, and ileum (Sikandar et al. 2017) as well as crypts in the caecum of broilers (Duangnumsawang et al. 2022). In the current study, the number of acidic GC increased by 4.5 times from day 7 to 35, whereas mixed GC number increased only by 1.5 times during 21 days of age and remained stable afterward. The density of acidic GC was also increased by around 2.6 times during the whole period of this study, while mixed GC density decreased with age. A greater number of GC, particularly acidic GC, may result in the production of more acidic mucins which appear to be less degradable by bacterial and host enzymes, thereby increasing resistance to pathogens and mechanical irritation (Montagne et al. 2004). Indeed, increasing the proportion of negatively charged (acidic) mucins alters physiochemical interactions between mucin molecules causing an increase in viscosity of the mucus layer, which may be associated with an age-related increase in gut bacterial diversity and bacterial-derived compounds (Liao et al. 2020). Modification of mucin molecules such as sialylation and sulfation, converts neutral mucins into acidic mucins and is reportedly promoted along with GC maturation (Hino et al. 2012). Thus, increased acidic mucins in the villus of ileum of current broilers may reflect GC maturation with age, which also enhance the protective property of intestinal mucus layer. According to Duangnumsawang et al. (2022), GC density, especially the acidic type was lower in the caecum (crypts) of broilers (used in the present study) compared with their ileum (villi). The observed variation in GC density in the ileum and caecum could be attributed to their morphology, physiological function and absorption capacity as well as bacterial number, composition and activity. In the ileum, GC population along villi secretes protective mucus layer to cover the epithelial surface while facilitating nutrients transportation from lumen to the underlying epithelium. In contrast, caecum acts as a fermentation chamber for microflora with higher bacterial number and activity than the ileum (Goodarzi Boroojeni et al. 2014), while nutrient absorption is not its main physiological function. Moreover, the mucus layer in the small intestine is usually thinner than the hindgut due to gut motility which propel digesta and mucus to the distal part of the intestine (Herath et al. 2020), thus may increase mucus renewal and stimulate GC proliferation in the ileum compared with the caecum.

## Conclusion

The present data demonstrated that age of broilers had a significant impact on microbial activity and immunological responses in the ileum, while the effect of probiotic or phytobiotic supplementation was totally absent. The genetic background of broilers, particularly their breed (Ross and Cobb), was found to have an effect on goblet cell count, certain bacterial metabolites, and cytokines expression in the ileum, while sex had almost no impact on these variables. A few interaction effects between the main factors were found on some of the investigated variables but they did not show meaningful biological patterns. This study was able to capture the alterations in microbial metabolites in the ileum of broilers at different ages which could potentially affect the development of gut morphology, goblet cell density, as well as expression of the cytokines and the proteins involved in epithelial barrier integrity. The observed age-related effects could be explained by the interaction between the gut microbiota and immune system and the direct effect of microbial metabolites on the gut morphology and cytokine response profile. Gut microbiota could affect maturation of the host immune system through its bioactive substances. However, further research is required to understand better the mechanisms behind the interaction between the host and its gut microbiota.

### Electronic supplementary material

Below is the link to the electronic supplementary material.

Supplementary Material 1

## Data Availability

All data generated or analyzed during this study are included in this published article and its supplementary information files.
