# Supplementary material for: Impact of feed additives and host-related factors on bacterial metabolites, mucosal integrity and immune response in the ileum of broilers
Source: Vet Res Commun. 2023 May 9;47(4):1861–78. doi: 10.1007/s11259-023-10135-9 (PMC10698104; doi:10.1007/s11259-023-10135-9)
Supplement: Supplementary file 1 — Supplementary Material 1 [file 11259_2023_10135_MOESM1_ESM.docx]

**SUPPLEMENTARY MATERIALS**

**Table 1A. *p*-values for the effect of interactions between main factors on ileal histomorphology***

| **Parameters** | ***p*-value** | | | | | | | | | | |
| --- | --- | --- | --- | --- | --- | --- | --- | --- | --- | --- | --- |
|  | **A*T** | **A*B** | **A*S** | **T*B** | **T*S** | **B*S** | **A*T*B** | **A*T*S** | **A*B*S** | **T*B*S** | **T*B*S*A** |
| ***Morphology ^1^*** |  |  |  |  |  |  |  |  |  |  |  |
| VH | 0.070 | 0.815 | 0.064 | 0.211 | 0.895 | 0.087 | 0.381 | 0.954 | 0.611 | 0.491 | 0.883 |
| VW | 0.908 | 0.852 | 0.450 | 0.997 | 0.493 | 0.197 | 0.696 | 0.696 | 0.439 | 0.842 | 0.490 |
| CD | 0.134 | 0.205 | 0.740 | 0.852 | 0.712 | 0.797 | 0.622 | 0.367 | 0.999 | 0.639 | 0.921 |
| V/C | 0.948 | 0.163 | 0.228 | 0.937 | 0.851 | 0.664 | 0.361 | 0.229 | 0.508 | 0.437 | 0.971 |
| VSA | 0.416 | 0.947 | 0.801 | 0.655 | 0.923 | 0.465 | 0.733 | 0.963 | 0.581 | 0.751 | 0.518 |
| ***Goblet cell number ^2^*** |  |  |  |  |  |  |  |  |  |  |  |
| Acidic | 0.960 | 0.506 | 0.779 | 0.491 | 0.133 | 0.691 | 0.267 | 0.212 | 0.964 | 0.374 | 0.689 |
| Mixed | 0.526 | 0.842 | 0.867 | 0.555 | 0.507 | 0.173 | 0.453 | 0.676 | 0.808 | 0.932 | 0.879 |
| Total | 0.346 | 0.377 | 0.955 | 0.435 | 0.916 | 0.147 | 0.471 | 0.979 | 0.711 | 0.857 | 0.741 |
| ***Goblet cell density ^3^*** |  |  |  |  |  |  |  |  |  |  |  |
| Acidic | 0.976 | 0.910 | 0.590 | 0.962 | 0.187 | 0.293 | 0.675 | 0.196 | 0.947 | 0.689 | 0.903 |
| Mixed | 0.418 | 0.618 | 0.144 | 0.868 | 0.232 | 0.089 | 0.811 | 0.248 | 0.926 | 0.574 | 0.832 |
| Total | 0.212 | 0.329 | 0.028 | 0.753 | 0.686 | 0.170 | 0.741 | 0.806 | 0.934 | 0.592 | 0.598 |

^1^ Villus height (VH), villus width (VW), crypt depth (CD) are measured in µm, V/C ratio was calculated by dividing villus height with crypt depth, villus epithelial surface area (VSA) was calculated by the multiplication of villus height and villus width, expressed as 10^3^ µm^2^

^2^ The average number of goblet cells per villus. Acidic represents the cells that are positive to Alcian blue dye. Mixed represents the cells that are positive to both Alcian blue and PAS dye. Total represents the sum of acidic and mixed goblet cells.

^3^ The average number of goblet cells per 100 µm villus height. Acidic represents the cells that are positive to Alcian blue dye. Mixed represents the cells that are positive to both Alcian blue and PAS dye. Total represents the sum of acidic and mixed goblet cells.

* The main factors consist of three age groups (A; day 7, 21 and 35 of age), three dietary treatments (T; control, probiotic and phytobiotic products), two breeds (B; Ross and Cobb) and two sexes (S; male and female). The trial consisted of 6 replicate-pens per treatment (40 birds per pen). Data were subjected to ANOVA to evaluate age, diet, breed and sex and Four-way ANOVA to evaluate their interactions using GLM procedure.

**Table 1B. The effect of interaction between age and sex on total goblet cell density in the ileum of broilers***

| **Age** | **Sex** | **Total goblet cell density ^1^** |
| --- | --- | --- |
| Day 7 | Male | 24.2 ± 0.69^abc^ |
|  | Female | 24.8 ± 0.73^ab^ |
| Day 21 | Male | 22.0 ± 0.68^bc^ |
|  | Female | 21.9 ± 0.54^c^ |
| Day 35 | Male | 21.7 ± 0.74^c^ |
|  | Female | 25.3 ± 0.78^a^ |

^1^ The average number of goblet cells per 100 µm villus height. Total represents the sum of acidic and mixed goblet cells.

^a,b,c^ Means ± SEM with different superscripts in a column differ significantly (*p =* 0.000).

* The trial was conducted with a 3 × 2 × 2 factorial arrangement of diet, breed and sex in a completely randomized design and consisted of 6 replicate-pens per treatment (40 birds per pen). Three age groups (day 7, 21 and 35 of age) and three dietary treatments (control, probiotic and phytobiotic products) were included in the analysis. The present results are reported as means of 36 replicate-pens. Means were separated by the Tukey's HSD post hoc test.

Table 2A. The effect of age, dietary treatment, breed and sex on metabolite concentration (µmol/g of fresh sample) in the ileum of broilers*

| **Parameters^1^** | **Age (A)** | | | **Treatment (T)** | | | **Breed (B)** | | **Sex (S)** | | **SEM** |
| --- | --- | --- | --- | --- | --- | --- | --- | --- | --- | --- | --- |
|  | **7** | **21** | **35** | **CO** | **PO** | **PY** | **Ross** | **Cobb** | **Male** | **Female** |  |
| ***Short chain fatty acid*** |  |  |  |  |  |  |  |  |  |  |  |
| Acetic acid | 2.40 ^b^ | 2.99 ^a^ | 2.16 ^b^ | 2.51 | 2.6 | 2.44 | 2.5 | 2.53 | 2.52 | 2.51 | 0.071 |
| Propionic acid | 0.14 ^a^ | 0.08 ^b^ | 0.12 ^ab^ | 0.12 | 0.11 | 0.12 | 0.11 | 0.12 | 0.11 | 0.12 | 0.006 |
| i-butyric acid | 0.11 ^a^ | 0.12 ^a^ | 0.00 ^b^ | 0.07 | 0.08 | 0.08 | 0.08 | 0.08 | 0.08 | 0.08 | 0.005 |
| n-butyric acid | 0.01 | 0.01 | 0.00 | 0.01 | 0.01 | 0.01 | 0.01 | 0.01 | 0.01 | 0.01 | 0.003 |
| i-valeric acid | 0.02 ^a^ | 0.01 ^b^ | 0.01 ^b^ | 0.01 | 0.01 | 0.01 | 0.01 | 0.01 | 0.01 | 0.01 | 0.002 |
| n-valeric acid | 0.02 ^b^ | 0.02 ^b^ | 0.03 ^a^ | 0.03 | 0.03 | 0.03 | 0.03 | 0.03 | 0.03 | 0.03 | 0.001 |
| Total SCFA ^2^ | 2.71 ^b^ | 3.24 ^a^ | 2.32 ^b^ | 2.74 | 2.84 | 2.69 | 2.73 | 2.78 | 2.76 | 2.76 | 0.076 |
| Total BCFA ^3^ | 0.13 ^a^ | 0.13 ^a^ | 0.01 ^b^ | 0.09 | 0.09 | 0.09 | 0.09 | 0.09 | 0.09 | 0.09 | 0.005 |
| ***Biogenic amine*** |  |  |  |  |  |  |  |  |  |  |  |
| Putrescine | 0.02 ^b^ | 0.02 ^b^ | 0.04 ^a^ | 0.03 | 0.02 | 0.03 | 0.02 | 0.03 | 0.03 | 0.02 | 0.003 |
| Histamine | 0.01 | 0.01 | 0.01 | 0.01 | 0.01 | 0.01 | 0.01 | 0.01 | 0.01 | 0.01 | 0.001 |
| Cadaverine | 0.01 ^b^ | 0.06 ^b^ | 0.13 ^a^ | 0.06 | 0.09 | 0.07 | 0.08 | 0.08 | 0.08 | 0.06 | 0.010 |
| Spermidine | 0.04^a^ | 0.03 ^b^ | 0.03 ^b^ | 0.03 | 0.03 | 0.03 | 0.03 | 0.03 | 0.04 | 0.03 | 0.001 |
| Spermine | 0.02 ^a^ | 0.01 ^b^ | 0.01 ^b^ | 0.01 | 0.01 | 0.01 | 0.01 | 0.01 | 0.01 | 0.01 | 0.001 |
| Total amine ^4^ | 0.24 ^b^ | 0.20 ^b^ | 0.62 ^a^ | 0.24 | 0.44 | 0.40 | 0.33 | 0.39 | 0.36 | 0.36 | 0.048 |
| ***Lactate*** |  |  |  |  |  |  |  |  |  |  |  |
| l- Lactic acid | 32.44 ^a^ | 19.00 ^b^ | 39.71 ^a^ | 26.53 | 33.67 | 30.91 | 31.73 | 29.01 | 30.93 | 29.82 | 1.592 |
| d‐Lactic acid | 12.89 ^a^ | 5.37 ^b^ | 13.54 ^a^ | 9.08 | 12.23 | 10.43 | 12.12 ^a^ | 9.01 ^b^ | 11.18 | 9.99 | 0.784 |
| Total lactic acids | 45.15 ^a^ | 24.36 ^b^ | 53.25 ^a^ | 35.61 | 45.89 | 41.19 | 43.84 | 37.94 | 42.01 | 39.81 | 2.295 |
| d‐ to l-lactic acid ratio | 0.29 ^a^ | 0.22 ^b^ | 0.29 ^a^ | 0.26 | 0.28 | 0.26 | 0.30 ^a^ | 0.24 ^b^ | 0.28 | 0.25 | 0.012 |
| ***Ammonium*** |  |  |  |  |  |  |  |  |  |  |  |
| NH_4_ | 3.21 ^a^ | 1.99 ^b^ | 2.10 ^b^ | 2.47 | 2.37 | 2.43 | 2.54 | 2.3 | 2.28 | 2.58 | 0.105 |

^1^ CO, Control; PO, Probiotic product; PY, Phytobiotic product

^2^ Total short chain fatty acid is the sum of acetate, propionate, i-butyrate, n-butyrate, i-valerate and n-valerate concentration.

^3^ Total branched chain fatty acid is the sum of i-butyrate and i-valerate concentration.

^4^ Total amine is the sum of putrescine, histamine, cadaverine, spermidine and spermine.

^a,b,c^ Means within a row of each main factors lacking a common superscript differ (*p* < 0.05).

* The trial was conducted with a 3 × 2 × 2 factorial arrangement of diet, breed and sex in a completely randomized design and consisted of 6 replicate-pens per treatment and 40 birds per pen. Data were subjected to ANOVA to evaluate age, diet, breed and sex and Four-way ANOVA to evaluate their interactions using GLM procedure.

**Table 2B. *p*-values for the effect of main factors and their interactions on ileal metabolite concentration of broilers***

| **Parameters** | ***p*-value** | | | | | | | | | | | | | | |
| --- | --- | --- | --- | --- | --- | --- | --- | --- | --- | --- | --- | --- | --- | --- | --- |
|  | **A** | **T** | **B** | **S** | **A*T** | **A*B** | **A*S** | **T*B** | **T*S** | **B*S** | **A*T*B** | **A*T*S** | **A*B*S** | **T*B*S** | **T*B*S*A** |
| ***Short chain fatty acid*** | |  |  |  |  |  |  |  |  |  |  |  |  |  |  |
| Acetic acid | <0.001 | 0.618 | 0.860 | 0.924 | 0.947 | 0.666 | 0.861 | 0.514 | 0.906 | 0.696 | 0.896 | 0.773 | 0.442 | 0.693 | 0.112 |
| Propionic acid | 0.001 | 0.761 | 0.318 | 0.390 | 0.519 | 0.244 | 0.623 | 0.874 | 0.705 | 0.894 | 0.951 | 0.599 | 0.681 | 0.805 | 0.893 |
| i-butyric acid | <0.001 | 0.364 | 0.800 | 0.960 | 0.775 | 0.835 | 0.335 | 0.579 | 0.732 | 0.484 | 0.499 | 0.652 | 0.814 | 0.169 | 0.612 |
| n-butyric acid | 0.090 | 0.355 | 0.286 | 0.512 | 0.428 | 0.333 | 0.689 | 0.609 | 0.336 | 0.754 | 0.298 | 0.448 | 0.855 | 0.417 | 0.658 |
| i-valeric acid | 0.009 | 0.845 | 0.778 | 0.671 | 0.474 | 0.076 | 0.584 | 0.807 | 0.833 | 0.035 | 0.373 | 0.574 | 0.754 | 0.419 | 1.000 |
| n-valeric acid | <0.001 | 0.800 | 0.366 | 0.837 | 0.883 | 0.465 | 0.713 | 0.829 | 0.884 | 0.038 | 0.491 | 0.914 | 0.862 | 0.794 | 0.506 |
| Total SCFA ^1^ | <0.001 | 0.725 | 0.768 | 0.999 | 0.905 | 0.597 | 0.921 | 0.589 | 0.873 | 0.677 | 0.875 | 0.834 | 0.532 | 0.707 | 0.142 |
| Total BCFA ^2^ | <0.001 | 0.447 | 0.941 | 0.872 | 0.644 | 0.617 | 0.335 | 0.538 | 0.906 | 0.632 | 0.405 | 0.961 | 0.984 | 0.288 | 0.750 |
| ***Biogenic amine*** |  |  |  |  |  |  |  |  |  |  |  |  |  |  |  |
| Putrescine | 0.007 | 0.853 | 0.074 | 0.064 | 0.918 | 0.080 | 0.056 | 0.797 | 0.750 | 0.242 | 0.999 | 0.912 | 0.261 | 0.565 | 0.257 |
| Histamine | 0.066 | 0.604 | 0.087 | 0.473 | 0.548 | 0.258 | 0.197 | 0.141 | 0.978 | 0.712 | 0.293 | 0.292 | 0.338 | 0.325 | 0.106 |
| Cadaverine | <0.001 | 0.708 | 0.522 | 0.378 | 0.817 | 0.861 | 0.274 | 0.767 | 0.533 | 0.981 | 0.570 | 0.801 | 0.765 | 0.918 | 0.295 |
| Spermidine | <0.001 | 0.967 | 0.269 | 0.081 | 0.211 | 0.257 | 0.315 | 0.760 | 0.436 | 0.010 | 0.513 | 0.347 | 0.691 | 0.741 | 0.386 |
| Spermine | <0.001 | 0.151 | 0.517 | 0.399 | 0.216 | 0.227 | 0.302 | 0.483 | 0.772 | 0.136 | 0.656 | 0.634 | 0.761 | 0.904 | 0.917 |
| Total amine ^3^ | <0.001 | 0.200 | 0.615 | 0.917 | 0.541 | 0.206 | 0.895 | 0.936 | 0.149 | 0.116 | 0.514 | 0.616 | 0.071 | 0.660 | 0.234 |
| ***Lactate*** |  |  |  |  |  |  |  |  |  |  |  |  |  |  |  |
| l- Lactic acid | <0.001 | 0.156 | 0.406 | 0.767 | 0.105 | 0.497 | 0.547 | 0.640 | 0.218 | 0.676 | 0.306 | 0.880 | 0.344 | 0.670 | 0.541 |
| d‐Lactic acid | <0.001 | 0.240 | 0.048 | 0.456 | 0.121 | 0.455 | 0.951 | 0.665 | 0.353 | 0.937 | 0.653 | 0.492 | 0.171 | 0.127 | 0.332 |
| Total lactic acids | <0.001 | 0.163 | 0.197 | 0.666 | 0.107 | 0.453 | 0.719 | 0.637 | 0.250 | 0.769 | 0.405 | 0.759 | 0.238 | 0.429 | 0.505 |
| d‐ to l-lactic acid ratio | 0.014 | 0.820 | 0.014 | 0.335 | 0.515 | 0.339 | 0.764 | 0.654 | 0.676 | 0.896 | 0.952 | 0.451 | 0.470 | 0.131 | 0.090 |
| ***Ammonium*** |  |  |  |  |  |  |  |  |  |  |  |  |  |  |  |
| NH_4_ | <0.001 | 0.882 | 0.254 | 0.084 | 0.994 | 0.016 | 0.376 | 0.506 | 0.845 | 0.995 | 0.598 | 0.922 | 0.769 | 0.499 | 0.184 |

^1^ Total short chain fatty acid is the sum of acetate, propionate, i-butyrate, n-butyrate, i-valerate and n-valerate concentration.

^2^ Total branched chain fatty acid is the sum of i-butyrate and i-valerate concentration.

^3^ Total amine is the sum of putrescine, histamine, cadaverine, spermidine and spermine.

*The main factors consist of three age groups (A; day 7, 21 and 35 of age), three dietary treatments (T; control, probiotic and phytobiotic products), two breeds (B; Ross and Cobb) and two sexes (S; male and female). The trial consisted of 6 replicate-pens per treatment (40 birds per pen). Data were subjected to ANOVA to evaluate age, diet, breed and sex and Four-way ANOVA to evaluate their interactions using GLM procedure.

Table 2C. The effect of interaction between breed and sex on spermidine concentration in the ileum of broilers*

| **Breed** | **Sex** | **Spermidine ^1^** |
| --- | --- | --- |
| Ross | Male | 0.041 ± 0.003^a^ |
|  | Female | 0.029 ± 0.003^b^ |
| Cobb | Male | 0.031 ± 0.003^ab^ |
|  | Female | 0.033 ± 0.003^ab^ |

^1^ The metabolite concentration is shown as μmol/g of fresh sample.

^a,b,c^ Means ± SEM with different superscripts in a column differ significantly (*p =* 0.009).

*The trial was conducted with a 3 × 2 × 2 factorial arrangement of diet, breed and sex in a completely randomized design and consisted of 6 replicate-pens per treatment (40 birds per pen). Two breeds (Ross and Cobb) and two sexes (male and female) were included in the analysis. The present results are reported as means of 18 replicate-pens. Means were separated by the Tukey's HSD post hoc test.

**Table 2D. The effect of interaction between age and breed on ammonium (NH_4_) concentration in the ileum of broilers***

| **Age** | **Breed** | **NH_4_ ^1^** |
| --- | --- | --- |
| Day 7 | Ross | 3.71 ± 0.508^a^ |
|  | Cobb | 2.66 ± 0.220^b^ |
| Day 21 | Ross | 1.87 ± 0.074^b^ |
|  | Cobb | 2.12 ± 0.090^b^ |
| Day 35 | Ross | 2.05 ± 0.092^b^ |
|  | Cobb | 2.16 ± 0.071^b^ |

^1^ The metabolite concentration is shown as μmol/g of fresh sample.

^a,b^ Means ± SEM with different superscripts in a column differ significantly (*p =* 0.000).

*The trial was conducted with a 3 × 2 × 2 factorial arrangement of diet, breed and sex in a completely randomized design and consisted of 6 replicate-pens per treatment (40 birds per pen). Three age groups (A; day 7, 21 and 35 of age) and two breeds (Ross and Cobb) were included in the analysis. The present results are reported as means of 36 replicate-pens. Means were separated by the Tukey's HSD post hoc test.

Table 3A. The impact of age, dietary treatment, sex and breed on expression of the genes (log_10_ gene expression^1^) related to epithelial barrier function and inflammatory markers of the ileum*

| **Parameters^2^** | **Age (A)** | | | **Treatment (T)** | | | **Breed (B)** | | **Sex (S)** | | **SEM** |
| --- | --- | --- | --- | --- | --- | --- | --- | --- | --- | --- | --- |
|  | **7** | **21** | **35** | **CO** | **PO** | **PY** | **Ross** | **Cobb** | **Male** | **Female** |  |
| *IL-1β* | -3.67^c^ | -1.20^a^ | -2.80^b^ | -2.58 | -2.50 | -2.59 | -2.6 | -2.51 | -2.57 | -2.54 | 0.073 |
| *IL-2* | -5.56^c^ | -4.03^a^ | -5.36^b^ | -4.99 | -4.98 | -4.93 | -4.96 | -4.97 | -4.98 | -4.96 | 0.056 |
| *IL-4* | -4.66^c^ | -1.93^a^ | -4.00^b^ | -3.56 | -3.52 | -3.52 | -3.58b | -3.48^a^ | -3.54 | -3.52 | 0.081 |
| *IL-6* | -5.26^b^ | -4.43^a^ | -5.56^c^ | -5.15 | -5.06 | -5.02 | -5.19^b^ | -4.97^a^ | -5.12 | -5.04 | 0.050 |
| *IL-8* | -3.10^b^ | -1.86^a^ | -3.28^c^ | -2.73 | -2.74 | -2.78 | -2.74 | -2.76 | -2.74 | -2.76 | 0.051 |
| *IL-10* | -6.21^c^ | -4.30^a^ | -5.43^b^ | -5.35 | -5.29 | -5.30 | -5.34 | -5.29 | -5.29 | -5.34 | 0.057 |
| *IL-12* | -4.44^c^ | -2.41^a^ | -3.61^b^ | -3.50 | -3.45 | -3.51 | -3.49 | -3.48 | -3.51 | -3.46 | 0.060 |
| *IL-17* | -3.51^b^ | -2.36^a^ | -4.15^c^ | -3.27 | -3.33 | -3.43 | -3.3 | -3.38 | -3.37 | -3.31 | 0.070 |
| *IL-18* | -3.13^c^ | -1.41^a^ | -2.73^b^ | -2.42 | -2.4 | -2.44 | -2.42 | -2.42 | -2.45 | -2.39 | 0.052 |
| *TNF-α* | -3.14^b^ | -1.85^a^ | -3.47^c^ | -2.81 | -2.81 | -2.83 | -2.87^b^ | -2.77^a^ | -2.81 | -2.83 | 0.050 |
| *IFN-γ* | -2.25^b^ | -0.69^a^ | -2.69^c^ | -1.87 | -1.86 | -1.89 | -1.84^a^ | -1.92^b^ | -1.91 | -1.84 | 0.062 |
| *TGF-β* | -1.29^c^ | 1.51^a^ | -1.11^b^ | -0.31 | -0.30 | -0.28 | -0.30 | -0.29 | -0.30 | -0.29 | 0.089 |
| *MUC2* | -0.32^c^ | 1.85^a^ | 0.11^b^ | 0.55 | 0.54 | 0.55 | 0.53 | 0.56 | 0.55 | 0.54 | 0.065 |
| *CLDN5* | -2.60^c^ | -0.27^a^ | -2.05^b^ | -1.64 | -1.65 | -1.62 | -1.67 | -1.61 | -1.64 | -1.64 | 0.069 |

^1^ log_10_ copy number per ng of RNA was calculated by dividing the copy number of targeted mRNA with the copy number of the housekeeping genes, converting values to the copy number per total RNA, and then transformed to log_10_ scale.

^2^ CO, Control; PO, Probiotic product; PY, Phytobiotic product; *IL*, interleukin; *TNF-α*, tumor necrosis factor alpha; *IFN-γ*, interferon gamma; *TGF-β2*, transforming growth factor beta 2; *CLDN5*, Claudin 5; and *MUC2*, Mucin 2

^a,b,^^c^ Means within a row of each main factors lacking a common superscript differ (*p* < 0.05).

* Results are reported as means of 6 replicate-pens. The trial was conducted with a 3 × 2 × 2 factorial arrangement of diet, breed and sex in a completely randomized design and consisted of 40 birds per pen. One bird per pen for each group were subjected to ANOVA using GLM procedure to evaluate age, diet, breed and sex.

**Table 3B*. p*-values for the effect of main factors and their interactions on gene expression in the caecum of broilers***

| **Parameters^1^** | ***p*-value** | | | | | | | | | | | | | | |
| --- | --- | --- | --- | --- | --- | --- | --- | --- | --- | --- | --- | --- | --- | --- | --- |
|  | **A** | **T** | **B** | **S** | **A*T** | **A*B** | **A*S** | **T*B** | **T*S** | **B*S** | **A*T*B** | **A*T*S** | **A*B*S** | **T*B*S** | **T*B*S*A** |
| *IL-1* | <0.001 | 0.039 | 0.083 | 0.670 | 0.971 | 0.920 | 0.062 | 0.947 | 0.701 | 0.400 | 0.194 | 0.929 | 0.909 | 0.514 | 0.672 |
| *IL-2* | <0.001 | 0.795 | 0.947 | 0.975 | 0.294 | 0.411 | 0.341 | 0.484 | 0.348 | 0.908 | 0.450 | 0.643 | 0.429 | 0.805 | 0.433 |
| *IL-4* | <0.001 | 0.425 | 0.016 | 0.976 | 0.855 | 0.578 | 0.590 | 0.565 | 0.581 | 0.324 | 0.650 | 0.769 | 0.275 | 0.824 | 0.481 |
| *IL-6* | <0.001 | 0.416 | 0.004 | 0.278 | 0.244 | 0.909 | 0.330 | 0.703 | 0.417 | 0.383 | 0.168 | 0.613 | 0.743 | 0.746 | 0.749 |
| *IL-8* | <0.001 | 0.748 | 0.703 | 0.719 | 0.932 | 0.824 | 0.181 | 0.278 | 0.496 | 0.678 | 0.443 | 0.854 | 0.949 | 0.823 | 0.856 |
| *IL-10* | <0.001 | 0.411 | 0.329 | 0.102 | 0.226 | 0.761 | 0.448 | 0.033 | 0.133 | 0.856 | 0.471 | 0.143 | 0.395 | 0.120 | 0.073 |
| *IL-12* | <0.001 | 0.106 | 0.856 | 0.299 | 0.085 | 0.702 | 0.638 | 0.121 | 0.575 | 0.273 | 0.482 | 0.162 | 0.999 | 0.839 | 0.077 |
| *IL-17α* | <0.001 | 0.433 | 0.372 | 0.471 | 0.782 | 0.740 | 0.335 | 0.815 | 0.071 | 0.921 | 0.244 | 0.851 | 0.593 | 0.052 | 0.598 |
| *IL-18* | <0.001 | 0.180 | 0.705 | 0.087 | 0.033 | 0.288 | 0.627 | 0.068 | 0.430 | 0.240 | 0.661 | 0.581 | 0.615 | 0.149 | 0.309 |
| *TNF-α* | <0.001 | 0.623 | 0.002 | 0.503 | 0.046 | 0.944 | 0.458 | 0.161 | 0.510 | 0.724 | 0.556 | 0.743 | 0.107 | 0.906 | 0.163 |
| *IFN-γ* | <0.001 | 0.655 | 0.031 | 0.145 | 0.482 | <0.001 | 0.459 | 0.737 | 0.599 | 0.813 | 0.464 | 0.371 | 0.258 | 0.394 | 0.137 |
| *TGF-β* | <0.001 | 0.844 | 0.789 | 0.912 | 0.531 | 0.634 | 0.866 | 0.250 | 0.910 | 0.316 | 0.716 | 0.843 | 0.438 | 0.533 | 0.091 |
| *MUC2* | <0.001 | 0.980 | 0.440 | 0.351 | 0.340 | 0.452 | 0.744 | 0.753 | 0.655 | 0.147 | 0.513 | 0.711 | 0.633 | 0.108 | 0.472 |
| *CLDN5* | <0.001 | 0.965 | 0.076 | 0.546 | 0.133 | 0.038 | 0.804 | 0.577 | 0.517 | 0.286 | 0.853 | 0.308 | 0.550 | 0.223 | 0.033 |

^1^ *IL*, interleukin; *TNF-α*, tumor necrosis factor alpha; *IFN-γ*, interferon gamma; *TGF-β2*, transforming growth factor beta 2; *CLDN5*, Claudin 5; and *MUC2*, Mucin 2

*The main factors consist of three age groups (A; day 7, 21 and 35 of age), three dietary treatments (T; control, probiotic and phytobiotic products), two breeds (B; Ross and Cobb) and two sexes (S; male and female). The trial consisted of 6 replicate-pens per treatment (40 birds per pen). Data were subjected to ANOVA to evaluate age, diet, breed and sex and Four-way ANOVA to evaluate their interactions using GLM procedure.

Table 3C. The effect of interaction between age and breed on *IFN-γ* gene expression in the ileum of broilers*

| **Age** | **Breed** | ***IFN-γ* ^1^** |
| --- | --- | --- |
| Day 7 | Ross | -2.31 ± 0.046^b^ |
|  | Cobb | -2.19 ± 0.051^b^ |
| Day 21 | Ross | -0.65 ± 0.041^a^ |
|  | Cobb | -0.72 ± 0.046^a^ |
| Day 35 | Ross | -2.54 ± 0.042^c^ |
|  | Cobb | -2.84 ± 0.060^d^ |

^1^ *IFN-γ*, interferon gamma; log_10_ copy number per ng of RNA was calculated by dividing the copy number of targeted mRNA with the copy number of the housekeeping genes, converting values to the copy number per total RNA, and then transformed to log_10_ scale.

^a,b,c,d^ Means ± SEM with different superscripts in a column differ significantly (*p =* 0.000).

*The trial was conducted with a 3 × 2 × 2 factorial arrangement of diet, breed and sex in a completely randomized design and consisted of 6 replicate-pens per treatment (40 birds per pen). Three age groups (A; day 7, 21 and 35 of age) and two breeds (Ross and Cobb) were included in the analysis. The present results are reported as means of 36 replicate-pens. Means were separated by the Tukey's HSD post hoc test.
